# Transcriptome and methylome changes in two contrasting mungbean genotypes in response to drought stress

**DOI:** 10.1186/s12864-022-08315-z

**Published:** 2022-01-25

**Authors:** Peilei Zhao, Bao Ma, Chunmei Cai, Jihua Xu

**Affiliations:** 1 College of Life Sciences, Qingdao Agricultural University, Qingdao, China; 2 College of Horticulture, Qingdao Agricultural University, Qingdao, China

**Keywords:** Drought stress, Transcriptome, Methylome, Mungbean, DNA methylation

## Abstract

**Background:**

Due to drought stress, the growth, distribution, and production of mungbean is severely restricted. Previous study combining physiological and transcriptomic data indicated different genotypes of mungbean exhibited variable responses when exposed to drought stress. Aside from the genetic variation, the modifications of environmentally induced epigenetics alterations on mungbean drought-stress responses were still elusive.

**Results:**

In this study, firstly, we compared the drought tolerance capacity at seedling stage by detecting physiological parameters in two contrasting genotypes wild mungbean 61 and cultivar 70 in response to drought stress. We found that wild mungbean 61 showed lower level of MDA and higher levels of POD and CAT, suggesting wild mungbean 61 exhibited stronger drought resistance. Transcriptomic analysis indicated totally 2859 differentially expressed genes (DEGs) were detected when 70 compared with 61 (C70 vs C61), and the number increased to 3121 in the comparison of drought-treated 70 compared with drought-treated 61 (D70 vs D61). In addition, when drought-treated 61 and 70 were compared with their controls, the DEGs were 1117 and 185 respectively, with more down-regulated DEGs than up-regulated in D61 vs C61, which was opposite in D70 vs C70. Interestingly, corresponding to this, after drought stress, more hypermethylated differentially methylated regions (DMRs) in 61 were detected and more hypomethylated DMRs in 70 were detected. Further analysis suggested that the main variations between 61 and 70 existed in CHH methylation in promoter. Moreover, the preference of methylation status alterations in D61 vs C61 and D70 vs C70 also fell in CHH sequence context. Further analysis of the correlation between DMRs and DEGs indicated in both D61 vs C61 and D70 vs C70, the DMRs in gene body was significantly negatively correlated with DEGs.

**Conclusions:**

The physiological parameters in this research suggested that wild mungbean 61 was more resistant to drought stress, with more hypermethylated DMRs and less hypomethylated DMRs after drought stress, corresponding to more down-regulated DEGs than up-regulated DEGs. Among the three DNA methylation contexts CG, CHG, and CHH, asymmetric CHH contexts were more dynamic and prone to be altered by drought stress and genotypic variations.

**Supplementary Information:**

The online version contains supplementary material available at 10.1186/s12864-022-08315-z.

## Background

Drought stress is one of the major environmental factors restricting crop growth, production, and distribution with more severe damage than other environmental stresses such as heat, low temperature and salinity stress [1–3]. Unlike animals, when subjected to drought stress, plants cannot escape but have to develop complicated defense systems, including a series of cellular, molecular, physiological, biochemical, anatomical and morphological responses [4]. For example, in water-deficient conditions, plants maintain cell turgor through osmotic adjustment to accumulate organic solutes such as glycine betaine, proline, and sugars to adjust water potential [5]. Meanwhile, plant have evolved detoxification systems to scavenge the excessive reactive oxidative species (ROS) caused by drought stress. The antioxidant pathways involve the enzymes superoxide dismutase (SOD), peroxidase (POD), catalase (CAT), and ascorbate peroxidase (APX) as well as the non-enzymatic compounds ascorbate, carotenoids, and glutathione [6].

When plants undergo drought stress, the stimuli induce signals transduction in multiple pathways, resulting in transcriptional changes of drought-responsive genes [7, 8]. With the popularization of next generation sequencing, RNA-seq is widely used to reveal the gene expression changes when exposed to drought stress, including drought-responsive transcription factors, plant hormone related genes, and protein kinases [9–12]. In the past decades, the genes associated with drought tolerance have been studied in detail, however drought tolerance is a complicated process not only involving transcriptional alterations but also genome-wide DNA methylation changes. DNA methylation, referred to as adding methyl group at the fifth position of cytosine pyrimidine ring, is a well-studied epigenetics mechanism [13, 14]. The methylated cytosines mainly happen in three DNA contexts, CG, CHG and CHH (where H = A, C or T) [15]. The maintenance of symmetrical methylation at CG and CHG occurs by DNA Methyltransferase 1 (MET1) and Chromomethylase 3 (CMT3) during DNA replication respectively [16, 17], while the maintenance of asymmetrical methylation at CHH is not template based but through RNA-directed DNA methylation (RdDM) with Domains Rearranged Methyltransferases 1 and 2 (DRM1, DRM2) [18].

DNA methylation as an important epigenetics marker involves in plant growth and development as well as plant abiotic stress tolerance and adaptations [19–21]. DNA methylation patterns in plants undergo dynamic changes depending on the tissues, plant species, and the specific type of stress [22]. Decreased DNA methylation was detected in root tissues of faba bean under drought stress, while increased DNA methylation was observed in root tissues of alfafa under salt stress [23, 24]. It has been clear that epigenetic modifications such as DNA methylation may affect gene expression and further contribute to phenotypic variation in response to environmental stress [25, 26]. Study in popular found genomic alterations of DNA methylation induced by drought stress could influence expression levels of related genes [27]. The obvious correlation between differentially DNA methylated regions and gene expression were detected when under drought stress in apple and mulberry [28, 29].

Mungbean (*Vigna radiata* L.) is an important fast-growing grain legume crop with rich protein, folate and iron, and is widely distributed in Asian countries especially India, China, Myanmar, and Indonesia [30, 31] . Mungbean can be processed into different food varieties such as sprout, flour, noodles, and porridge, which provide nutrition and tasty flavor for human beings [32]. However, due to abiotic and biotic stress, the yield of mungbean is low, and drought stress restrictions on mungbean production is becoming more severe [33]. Modern mungbean cultivars were derived from domestication and selection of the original mungbean species in India [34]. Normally, wild species contain valuable genes and resources which tend to be disappeared in the process of domestication and selection in breeding [35, 36]. Studies indicated wild soybean possessing multiple valuable candidate genes endowing the plants with stronger tolerance to drought stress [37, 38]. In addition, wild mungbean (TC1996) has shown complete bruchid resistance compared with cultivated mungbean [39].

In this study, we compared the drought tolerance capacity at seedling stage by detecting physiological parameters in two contrasting mungbean genotypes in response to drought stress. The comparative transcriptome analysis integrated with methylome study aimed to reveal the DNA methylation pattern and gene expression variations in control and drought-stressed conditions between the two contrasting genotypes, which might provide clues for.

the potential use of wild germplasm as a drought-tolerant resource in mungbean cultivar breeding.

## Results

### Comparison of physiological parameters of two mungbean genotypes exposed to drought stress

**Fig. 1:**
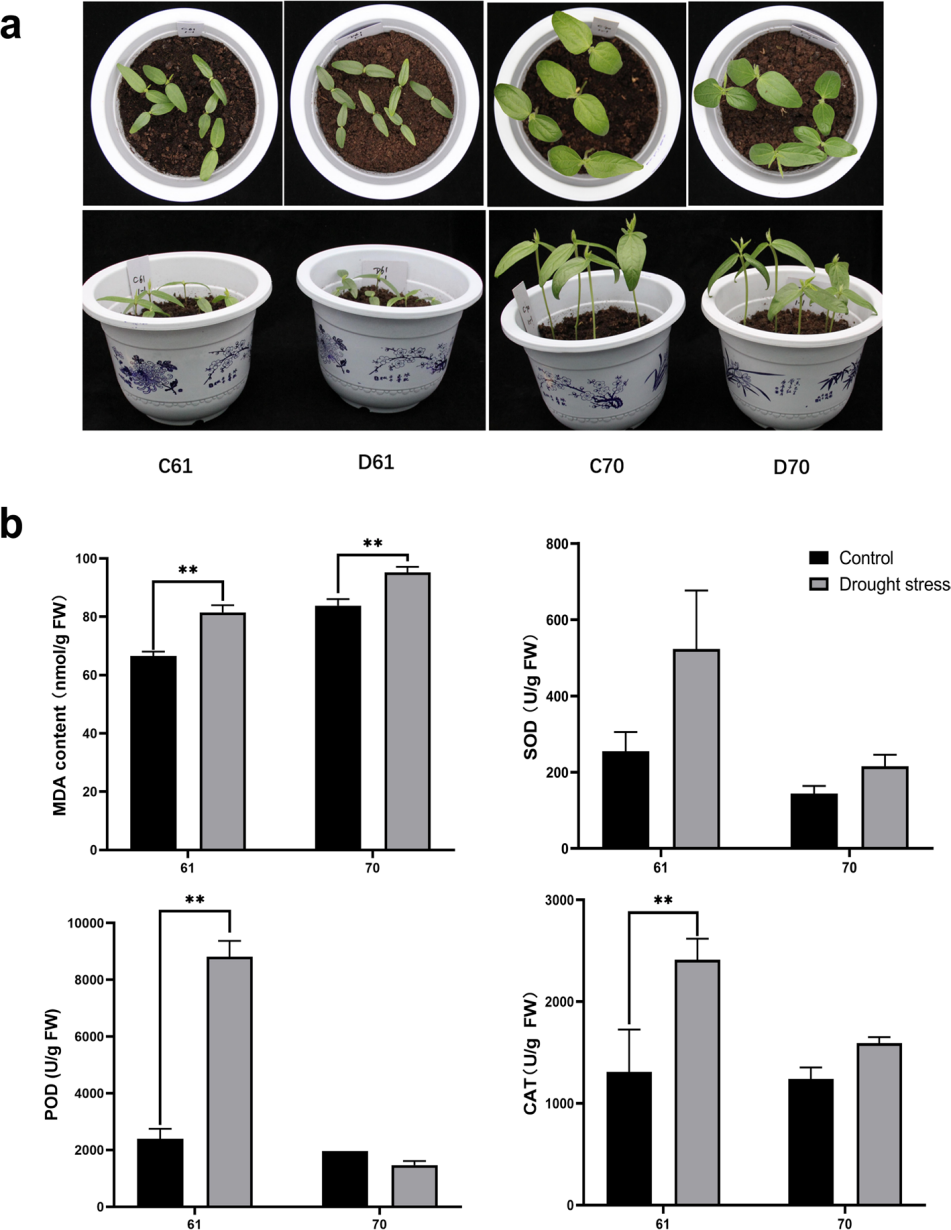
Phenotypic and physiological parameters changes in two mungbean genotypes under drought stress. **a** Phenotypes of two mungbean genotypes plants in control and drought-stressed conditions. C61 and D61 mean well-watered and drought-stressed wild mungbean 61; C70 and D70 mean well-watered and drought-stressed mungbean cultivar 70. **b** MDA, SOD, POD, and CAT content of two mungbean genotypes plants in control and drought-stressed conditions. ** represents *p* < 0.01

C61 and C70, representing control of wild and cultivated mungbean plants, were well-watered, while D61 and D70 plants were drought-stressed. It was obvious after drought treatment, D61 and D70 plants exhibited phenotypic changes, such as small stature and lower height (Fig. 1a). Leaf samples were collected from the vegetative 1 (V1) stage seedlings of the two contrasting mungbean plants for physiological parameters determination. We measured the content of malondialdehyde (MDA), which is the biomarkers of oxidative stress [12], and the antioxidant-related enzymes including SOD, POD, and CAT (Fig. 1b). Compared with control, the MDA content was significantly increased in both drought-stressed genotypes (*p* < 0.01) (Fig. 1b). However, in C70 and D70, the accumulation of MDA was higher than the levels in C61 and D61. The SOD level was also increased in drought-stressed condition in both genotypes but not statistically significant. Interestingly, both the content of POD and CAT in D61 was significantly higher than C61 (*p* < 0.01) but there was no significant difference between C70 and D70. These data suggested wild mungbean genotype 61 presented stronger resistance when subjected to drought stress.

### Transcriptomic changes of two mungbean genotypes in response to drought stress

**Fig. 2:**
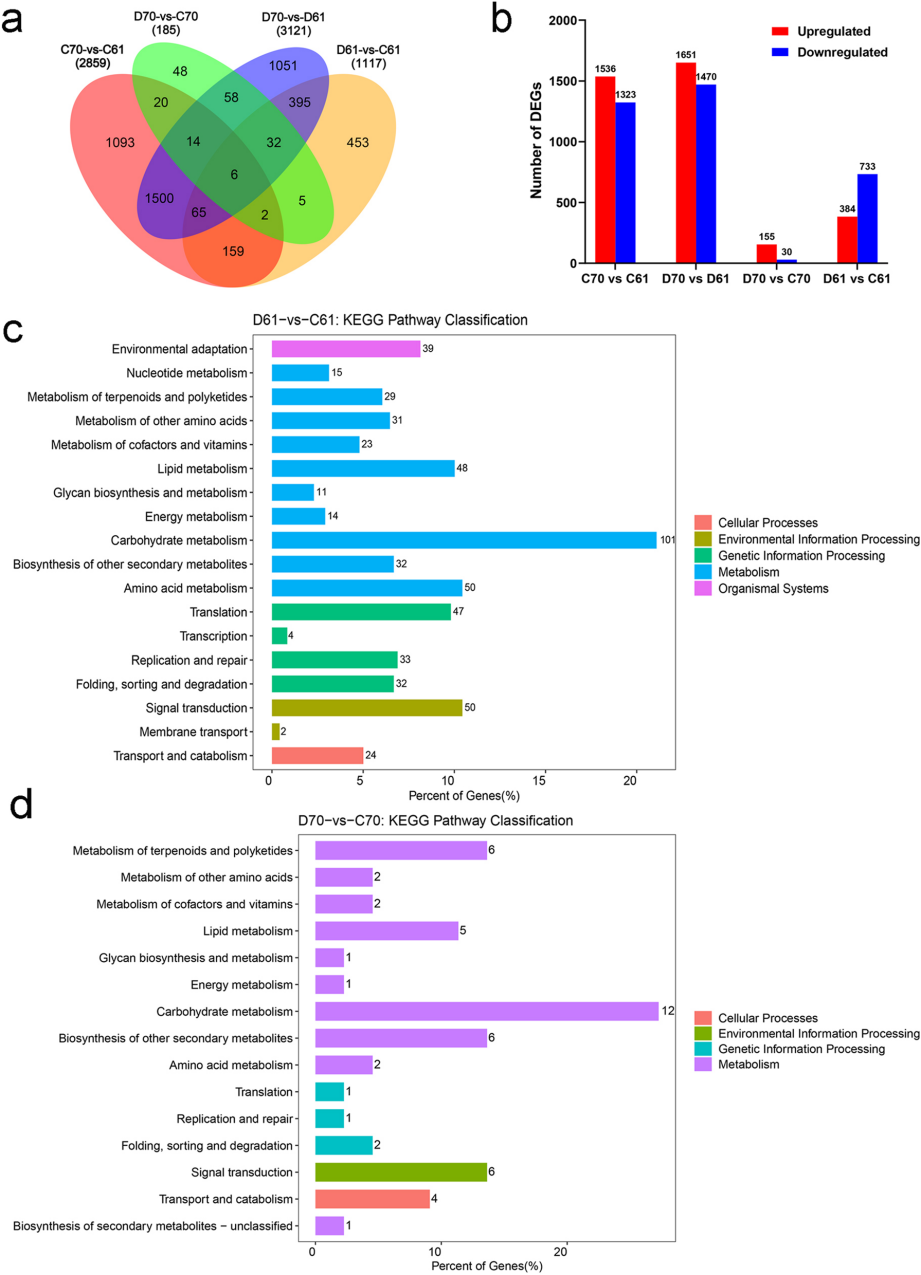
Differentially expressed genes in different comparisons and the KEGG pathway analysis. **a** Venn diagrams of DEGs in four pairwise comparisons. **b** Numbers of upregulated and downregulated DEGs in four pairwise comparisons. KEGG pathway enrichments of DEGs in D61 vs C61 (**c**) and D70 vs C70 (**d**). D61 vs C61, drought-stressed wild mungbean 61 versus well-watered; D70 vs C70, drought-stressed cultivar mungbean 70 versus well-watered

To further reveal the molecular bases accounted for the different performance of two genotypes when exposed to drought stress, the RNA-Seq analysis was conducted in the two genotypes in control and drought stress conditions. Among all eight samples investigated, the raw reads generated were between 40.53 million and 48.09 million, with more than 97% valid bases (Table 1). The Q30 of all eight samples was consistently over 94% (Table 1). These parameters indicated the sequencing data quality was high and could be used for further analysis. From the four pairwise comparisons, it was obvious that the number of differentially expressed genes (DEGs) was the most in D70 vs D61 comparison group, followed by C70 vs C61 (Fig. 2a), among the DEGs in these two groups, there were more upregulated genes than downregulated genes (Fig. 2b, Additional file 1: Fig. S1 ab). Totally 1117 DEGs were identified after drought stress compared with control in wild mungbean 61 (D61 vs C61), with 384 upregulated and 733 downregulated. However, only 185 DEGs were found in D70 vs C70, with 155 upregulated and 30 downregulated (Fig. 2b, Additional file 1: Fig. S1 cd). In order to validate the accuracy of gene expression data generated by RNA-Seq, we selected eight DEGs, most of which were transcription factors related to drought stress, and conducted qRT-PCR assays. The results indicated the trends of relative expression level of upregulated and downregulated genes of qRT-PCR were similar to that calculated by transcriptome sequencing (Additional file 2: Fig. S2), confirming the reliability of transcriptome data. Kyoto Encyclopedia of Genes and Genomes (KEGG: http://www.genome.jp/kegg/) analysis of DEGs in D61 vs C61 and D70 vs C70 was performed [40]. The results indicated that the highest number of transcripts were enriched in carbohydrate metabolism and signal transduction pathways, followed by amino acid metabolism and lipid metabolism (Fig. 2c, d).

**Table 1:** Statistics of RNA-seq for control and drought-treated samples of wild mungbean 61 and mungbean cultivar 70

Samples	Raw reads (M)	Raw bases (G)	Clean reads (M)	Clean bases (G)	Valid bases (%)	Q30	GC
						(%)	(%)
C61–1	43.18	6.48	42.89	6.41	98.9	95.12	45.29
C61–2	43.90	6.58	43.58	6.51	98.84	95.22	45.48
C70–1	47.10	7.07	46.39	6.88	97.36	94.49	44.64
C70–2	40.53	6.08	39.91	5.92	97.32	94.43	44.6
D61–1	41.86	6.28	41.56	6.21	98.83	95.22	45.56
D61–2	48.09	7.21	47.87	7.16	99.24	95.27	44.93
D70–1	43.62	6.54	42.97	6.36	97.16	94.57	44.68
D70–2	46.42	6.96	45.76	6.76	97.10	94.44	42.49

C61–1, C61–2 and C70–1, C70–2 represent two replicates of well-watered wild mungbean 61 and mungbean cultivar 70, respectively; D61–1, D61–2 and D70–1, D70–2 represent two replicates of drought-stressed wild mungbean 61 and mungbean cultivar 70, respectively. Raw reads/bases: reads/bases generated by Illumina HiSeq X Ten platform. Clean reads/bases: reads/bases after filtering poor quality score reads and trimming adaptors using Trimmomatic v0.32 program. Valid bases (%) = (Clean bases number / Raw bases number) * 100%. Q30: Phred quality score of 30; GC: GC content

### Methylome profiles in wild and cultivated mungbean

**Fig. 3:**
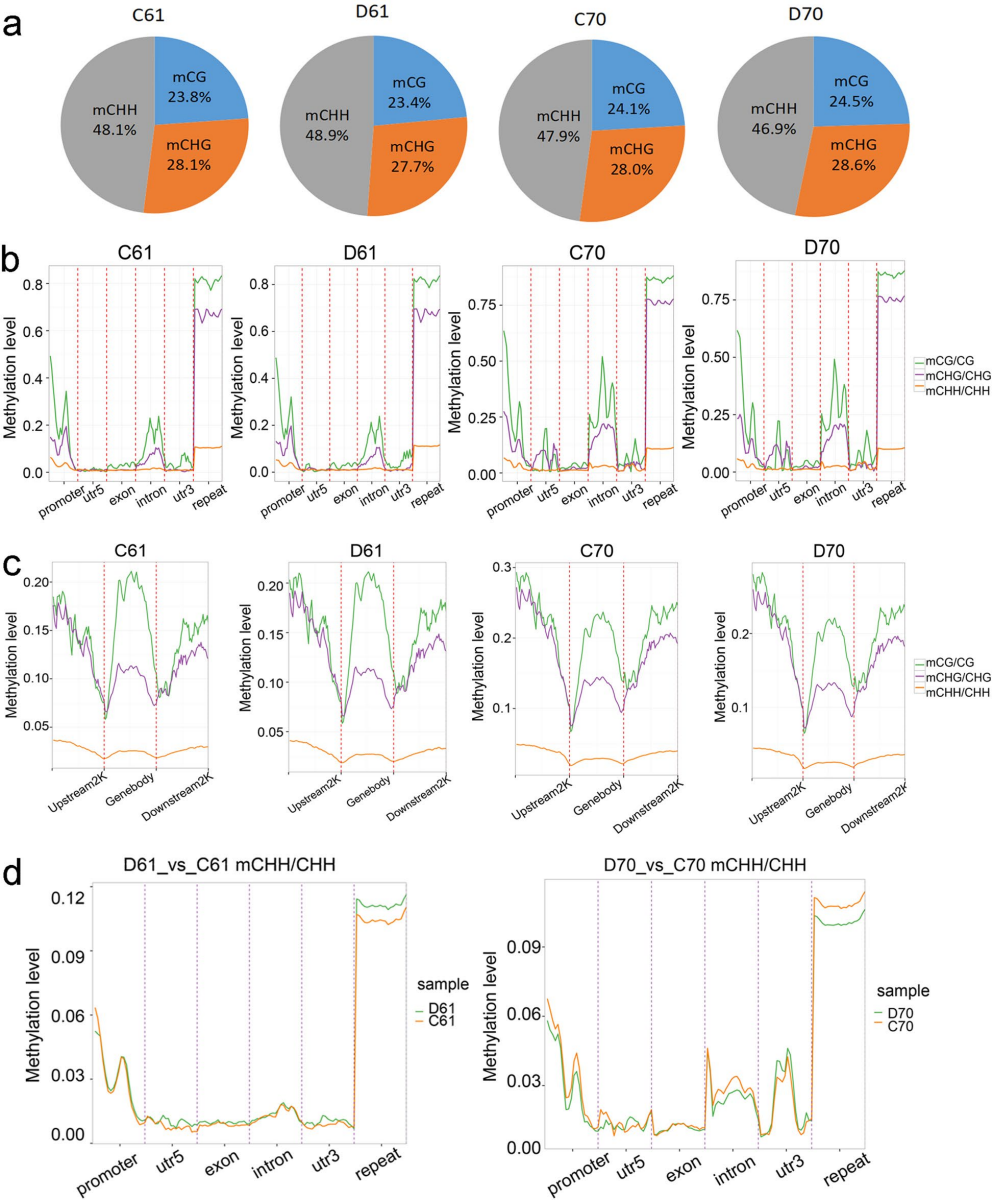
Methylation profiles in two mungbean genotypes. **a** The relative proportion of mCGs, mCHGs, and mCHHs in two mungbean genotypes in control and drought-stressed conditions. The level of methylation in different gene features (**b**) and gene body, upstream 2 K and downstream 2 K regions (**c**). The comparison of methylation level in different gene features in D61 vs C61 and D70 vs C70 (**d**). C61 and D61 mean well-watered and drought-stressed wild mungbean 61; C70 and D70 mean well-watered and drought-stressed mungbean cultivar 70

In total, whole-genome bisulfite sequencing (WGBS) results generated 71.9–82.7 million raw reads for each sample (Table 2). After filtering the low-quality data, 70.1–80.6 million clean reads were mapped to the reference genome using Bismark software. The mapping rate ranged from 56.84 to 79.08% (Table 2). For each sodium bisulfite treated library, the unmethylated lambda DNA was used as reference for conversion rate calculation. The results showed that the conversation efficiency was over 99% in all of the samples (Table 2). Genome-wide screening revealed that 18,227,407 methylated cytosines were detected in C61, the proportion of methylated CG, CHG and CHH was 23.8, 28.1, and 48.1% respectively (Fig. 3a). After drought treatment, there was a slight change in the methylated cytosines proportions in three sequence contexts, with CHH methylation increased to 48.9%, CG and CHG methylation decreased to 23.4 and 27.7%. By contrast, the number of methylated cytosines in C70 (30,417,748) and D70 (29,403,037) was more than in wild mungbean, but the proportions of methylated CG, CHG, and CHH was similar to wild mungbean. Interestingly, the percentage of methylated CHH decreased from 47.9 to 46.9% after drought treatment in cultivated mungbean70 (Fig. 3a).

**Table 2:** Statistics of WGBS for control and drought stress treated samples of wild mungbean 61 and mungbean cultivar 70

Samples	Raw reads (M)	Raw bases (G)	Clean reads (M)	Clean bases (G)	Valid bases (%)	Mapped reads (M)	Mapping rate (%)	BS conversion rate (%)
C61–1	78.39	23.52	76.55	20.92	88.95	43.76	57.16	99.23
C61–2	77.49	23.25	75.55	20.61	88.65	43.24	57.23	99.27
D61–1	77.89	23.37	76.07	20.78	88.92	43.45	57.12	99.14
D61–2	79.49	23.85	77.58	21.19	88.85	44.10	56.84	99.24
C70–1	82.71	24.81	80.64	21.94	88.43	63.43	78.66	99.21
C70–2	71.94	21.58	70.12	19.14	88.69	55.45	79.08	99.25
D70–1	72.69	21.81	70.85	19.34	88.67	55.94	78.95	99.32
D70–2	73.02	21.91	71.37	19.50	89.00	56.23	78.79	99.34

C61–1, C61–2 and C70–1, C70–2 represent two replicates of well-watered wild mungbean 61 and mungbean cultivar 70, respectively; D61–1, D61–2 and D70–1, D70–2 represent two replicates of drought-stressed wild mungbean 61 and mungbean cultivar 70, respectively. Raw reads/bases: reads/bases generated by Illumina Novaseq platform. Clean reads/bases: reads/bases after filtering poor quality score reads and trimming using fastp software. Valid bases (%) = (clean reads number / raw reads number) * 100%. Mapping rate = (mapped reads / clean reads) * 100%. BS conversion rate (%) = (converted cytosines / total cytosines in unmethylated lambda DNA reference) * 100%

DNA methylation patterns in CG, CHG, and CHH were also analyzed in different mungbean genomic regions as well as gene body, promoter, and downstream 2 K region (Fig. 3b,c). It was observed CG methylation was the highest across genomic regions, followed by CHG and CHH. CG and CHG methylation level showed similar trends, for example, in wild mungbean 61 high methylation was observed in promoter and it decreased in 5UTR, increased in intron and decreased again in 3UTR (Fig. 3b). The repeat region showed the highest level of methylation (Fig. 3b). In wild mungbean 61, the highest CG methylation level was observed in upstream 2 K and gene body, followed by downstream 2 K, whereas in mungbean cultivar 70 the highest methylation level was found in upstream 2 K, followed by gene body and downstream 2 K (Fig. 3c). The tends of CHG and CHH methylation changes was similar between mungbean 61 and 70 (Fig. 3c). In addition, it was obvious in D61 vs C61, the increase of CHH was mainly contributed by 5UTR, exon, 3UTR, and repeat regions, while in D70 vs C70, the decrease of CHH was mainly contributed by promoter, intron, and repeat regions (Fig. 3a,d).

### Differentially methylated regions in wild and cultivated mungbean

**Fig. 4:**
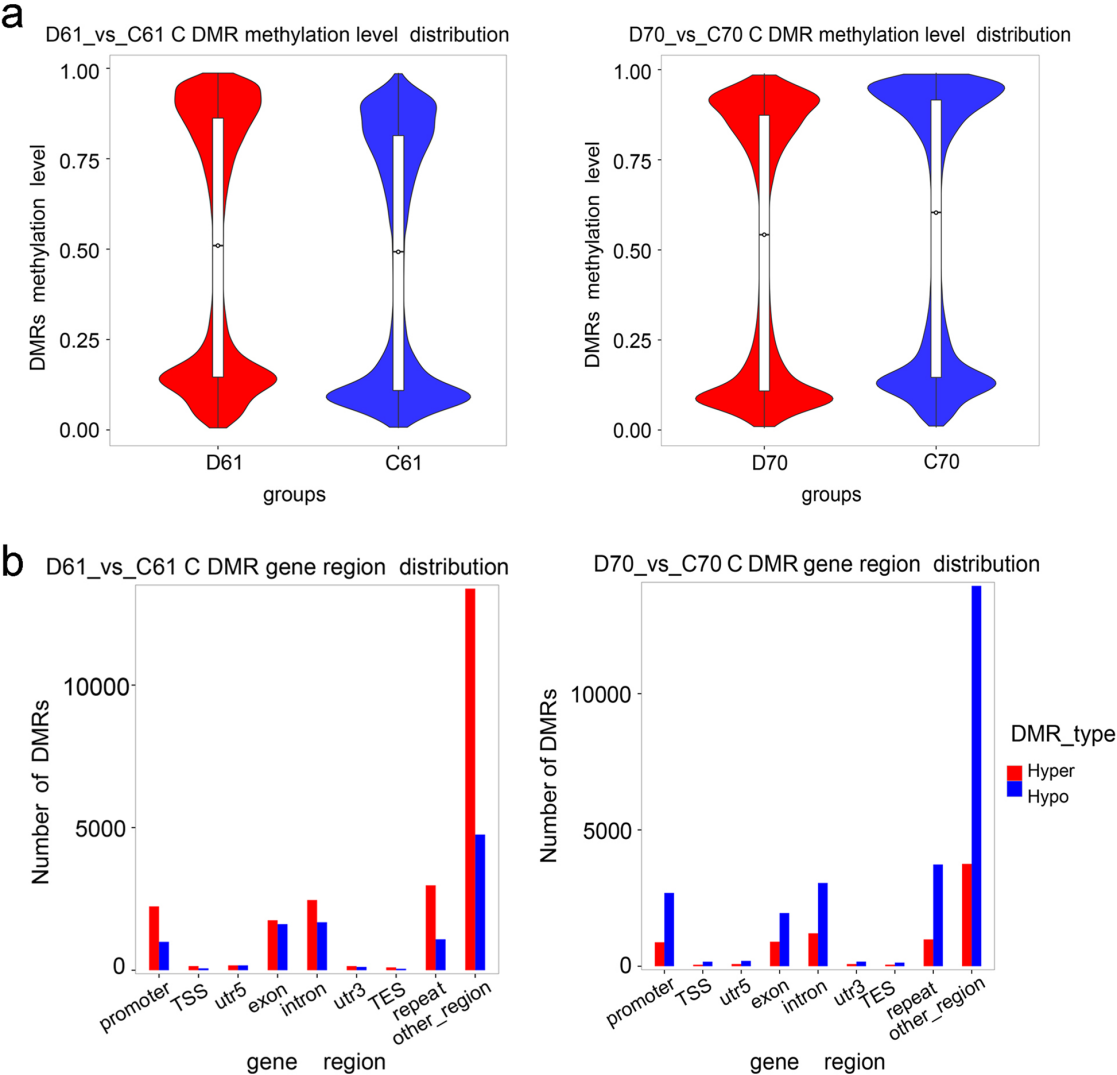
Differentially methylated regions distribution in D61 vs C61 and D70 vs C70. **a** Methylation level distribution of differentially methylated regions (DMRs) by violin boxplots. **b** Number of DMRs in different regions across genome. D61 vs C61, drought-stressed wild mungbean 61 versus well-watered; D70 vs C70, drought-stressed cultivar mungbean 70 versus well-watered

**Fig. 5:**
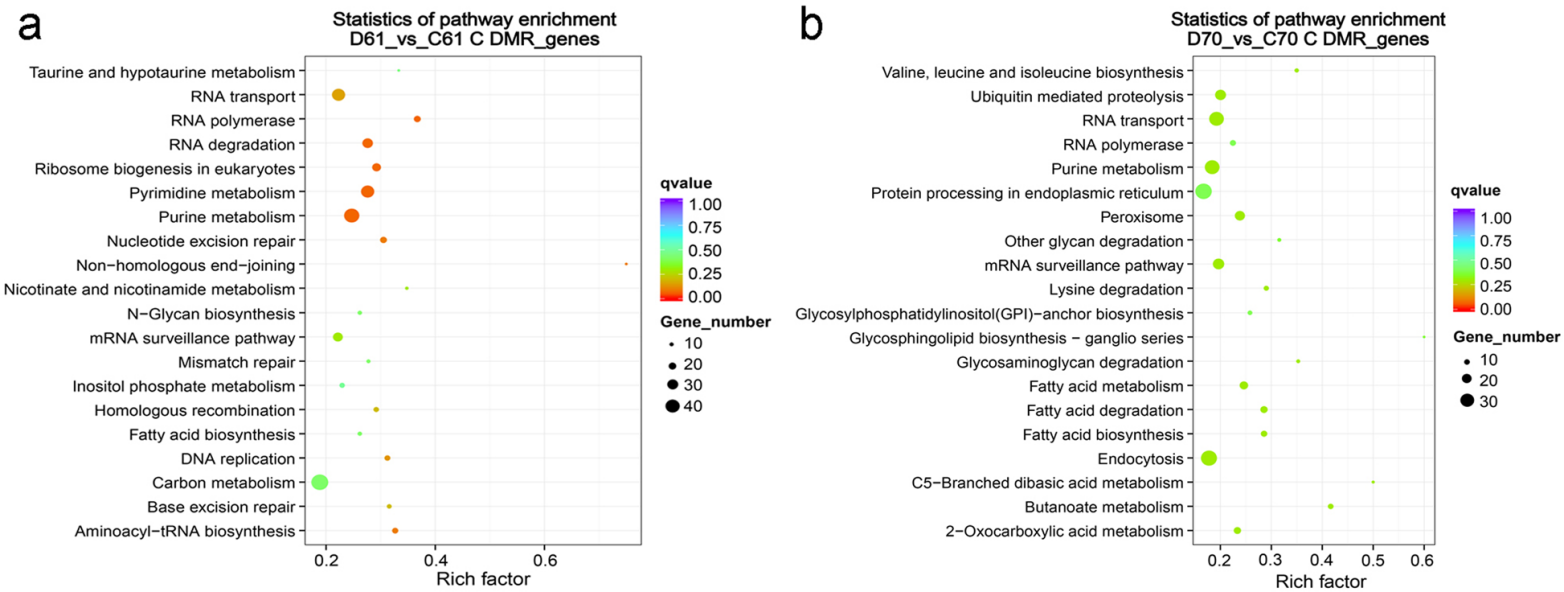
KEGG pathway enrichment of differentially methylated genes. D61 vs C61, drought-stressed wild mungbean 61 versus well-watered; D70 vs C70, drought-stressed cultivar mungbean 70 versus well-watered

We further compared the differentially methylated regions (DMRs) between wild and cultivated mungbean in control and drought stress conditions. Totally, we identified 12,111 hypermethylated and 6578 hypomethylated DMRs in the wild mungbean D61 vs C61, while in the cultivar mungbean D70 vs C70, the number of hypermethylated DRMs was only 4988 and hypomethylated DMRs was 14,747 (Additional file 3: Fig. S3). After drought stress, increased methylation level of DMRs in wild mungbean 61 were detected in all CG, CHG, and CHH contexts, especially in CHH context (Fig. 4a, Additional file 4: Fig. S4a). On the contrary, the decreased methylation level of DMRs were detected in D70 vs C70 in all CG, CHG, and CHH contexts, especially in CHH context (Fig. 4a, Additional file 4: Fig. S4b). Further detailed comparative analysis related to the genome-wide distribution of DMRs was conducted (Fig. 4b, Additional file 4: Fig. S4c,d). Overall, the hypermethylated DMRs or hypomethylated DMRs in D61 vs C61 and D70 vs C 70 were mainly distributed in promoter, exon, intron and repeat regions (Fig. 4b). Further analysis indicated that the main hypermethylated DMRs after drought stress in wild mungbean 61 were distributed in promoter and intron in CG; promoter, exon and intron in CHG; and promoter, intron and repeat in CHH context (Additional file 4: Fig. S4c). By contrast, the hypomethylated DMRs in mungbean 70 after drought stress mainly distributed in promoter, exon, intron, and repeat regions in all three DNA contexts (Additional file 4: Fig. S4d). The KEGG pathway analysis was conducted in order to investigate the associated biological functions and pathways of the DMRs. The results indicated in D61 vs C61, the DMRs were mainly distributed in pathways such as purine metabolism, RNA transport, pyrimidine metabolism, RNA degradation and carbon metabolism (Fig. 5a). The first two pathways were also observed in the enrichment of D70 vs C70, in addition, protein processing in endoplasmic reticulum and endocytosis were also enriched in D70 vs C70 (Fig. 5b).

### Relationship between DNA methylation status and gene expression levels

**Fig. 6:**
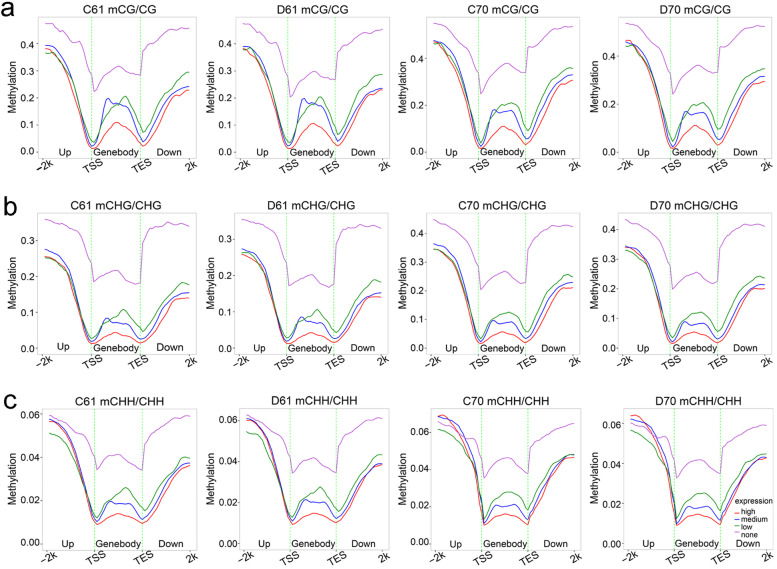
Relationship between gene expression and DNA methylation in C61, D61, C70 and D70. DNA methylation levels distributions in upstream 2 K, gene body, and downstream 2 K by different expression levels at CG (**a**), CHG (**b**), and CHH (**c**) DNA contexts. C61 and D61 mean well-watered and drought-stressed wild mungbean 61; C70 and D70 mean well-watered and drought-stressed mungbean cultivar 70

**Fig. 7:**
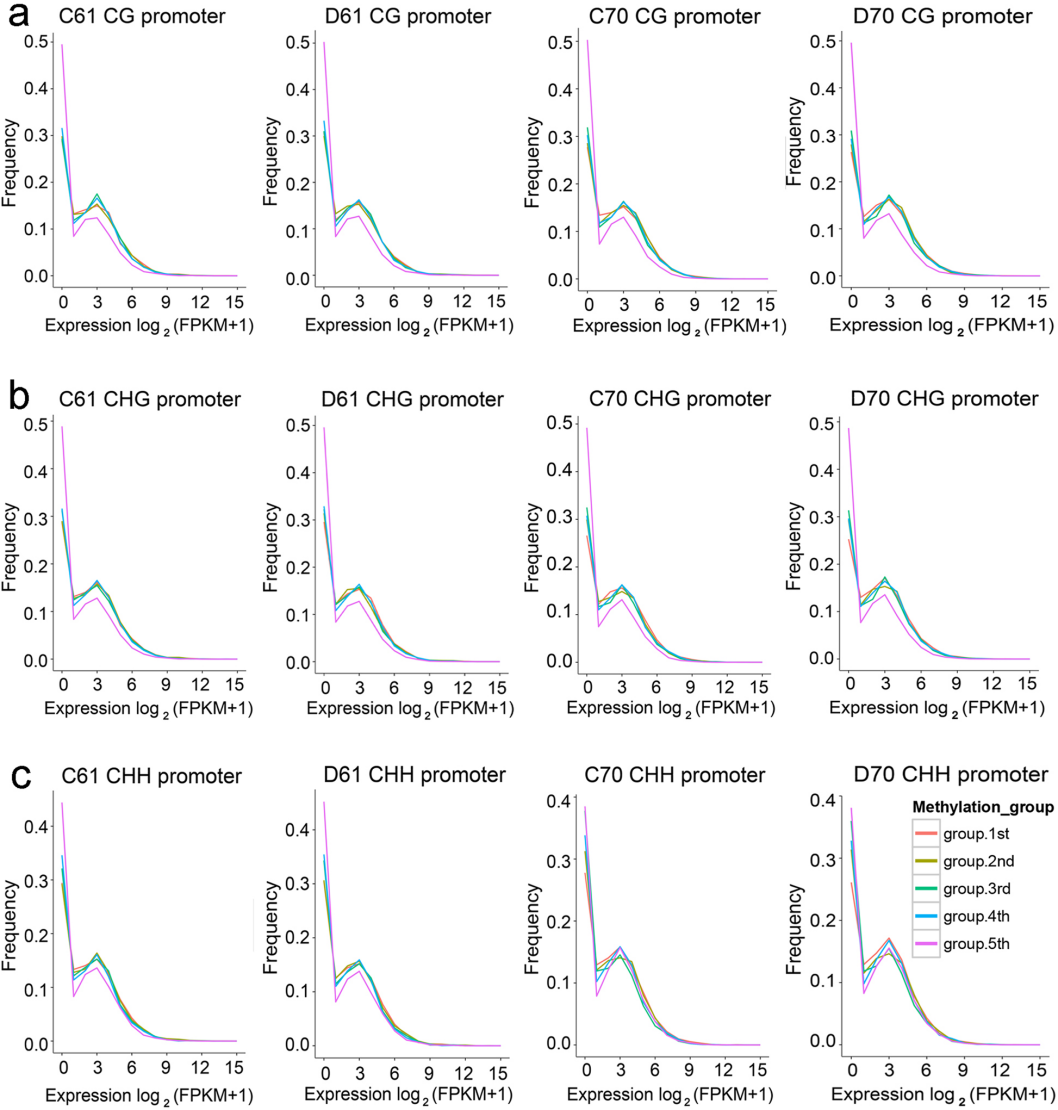
Relationship between DNA methylation and gene expression in C61, D61, C70 and D70 in promoter. Expression profiles of different methylated levels at CG (**a**), CHG (**b**) and CHH (**c**) were investigated. The promoter methylation levels were classified into five groups with group.1st the lowest and group.5th the highest. C61 and D61 mean well-watered and drought-stressed wild mungbean 61; C70 and D70 mean well-watered and drought-stressed mungbean cultivar 70

In order to investigate whether DNA methylation is regulated by gene expression, the correlation analysis of gene expression and DNA methylation was conducted. As predicted, the unexpressed genes had the highest methylation level in all gene body, promoter, and downstream 2-kb region in CG and CHG sequence contexts (Fig. 6a,b). While the lowest methylation was detected in the genes showed high expression in all regions of CG methylation and in gene body and downstream 2-kb region of CHG methylation (Fig. 6a,b). In contrast, for CHH methylation, the unexpressed genes showing the highest methylation level was observed in all regions in the wild mungbean 61, but only in gene body and downstream 2-kb region in cultivar mungbean 70 (Fig. 6c). In addition, the low expressed genes had the lowest CHH methylation level in promoter, whereas in gene body and downstream 2-kb region, the high expressed genes had the lowest CHH methylation level (Fig. 6c). We further studied relationship between DNA methylation and gene expression. Based on methylation levels, the methylated genes were divided into five groups with group first the lowest methylation level and group fifth the highest methylation level (Fig. 7**,** Additional file 5: Fig. S5). We found that in promoter, genes with the highest methylation levels showed the lowest expression levels in all three DNA sequence contexts in the wild mungbean 61, but only in CG and CHG in cultivar mungbean 70 (Fig. 7). In gene body, genes with the highest methylation levels showed the lowest expression levels in all CG, CHG and CHH (Additional file 5: Fig. S5), and moderately CG methylated genes showed the highest level of expression (Additional file 5: Fig. S5a).

### Differentially methylated regions and related differentially expressed genes

**Fig. 8:**
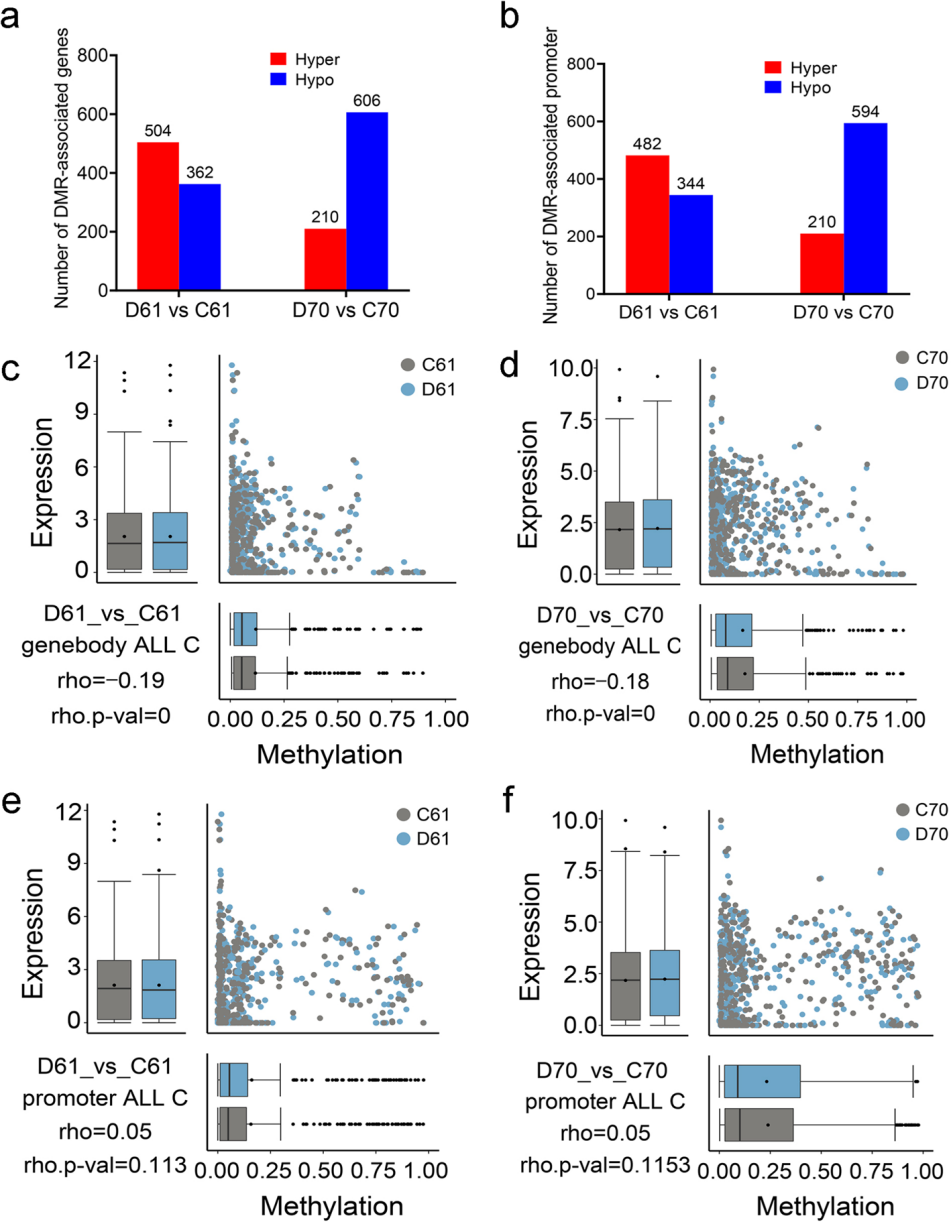
Differentially methylated regions and related differentially expressed genes. Differentially expressed genes (DEGs) identified as hyper- and hypomethylated differentially methylated regions (DMRs)-associated genes (**a**) and promoters (**b**). Relationship between DMRs in gene body and DEGs in D61 vs C61 (**c**), and D70 vs C70 (**d**). Relationship between DMRs in promoter and DEGs in D61 vs C61 (**e**), and D70 vs C70 (**f**). D61 vs C61, drought-stressed wild mungbean 61 versus well-watered; D70 vs C70, drought-stressed cultivar mungbean 70 versus well-watered

In order to study the global effect of DRMs on related gene expression, we analyzed the DMRs related genes and promoters. As a result, we found in D61 vs C61 there were 504 and 362 DEGs identified as hyper- and hypomehylated DMR-associated genes, while in D70 vs C70 the corresponding DEGs number were 210 and 606 DEGs respectively (Fig. 8a). Similarly, 482 and 344 DEGs were detected as hyper- and hypomehylated DMR-associated promoters in D61 vs C61. In contrast, in D70 vs C70 there were 210 and 594 hyper- and hypomehylated DMR-associated promoters identified (Fig. 8b). The Spearman rank correlation coefficient was used to test associations between DMRs and DEGs, and Spearman’s rho was used as a measure for correlation. The results indicated in D61 vs C61, the gene body methylation was negatively correlated with gene expression (Spearman rho = − 0.19, *p* value = 0) (Fig. 8c). Similar result was detected in cultivar mungbean D70 vs C70 (Spearman rho = − 0.18, p value = 0) (Fig. 8d). However, there were no clear correlation between promoter methylation and gene expression in both D61 vs C61 and D70 vs C70 (Fig. 8e,f). Altogether, the results suggested DNA methylation could partially explain the differential transcript abundances of related genes.

## Discussion

In this research, firstly, we compared the responses of two mungbean genotypes after drought stress treatment at seedling stage based on physiological parameters. Recent study indicated variable responses to drought stress among different mungbean varieties according to physiological data and transcriptomic study [33]. Aside from the genetic variation, it was reported environmentally induced epigenetics alterations also could modify stress response and broaden plant phenotypic variation [26]. Therefore, in this research, we integrated physiological parameters with transcriptome and whole genome bisulfite sequencing analysis to reveal the molecular mechanism which might explain the different performance of the two genotypes when exposed to drought stress.

After drought treatment at seedling stage, the two genotypes showed visible differences compared to control. We further found that wild mungbean 61 showed significantly lower level of MDA and higher levels of POD and CAT. As lower level of MDA and increased antioxidant enzymes activity are correlated with cell membrane stability and enhanced antioxidant defense system, which could protect plants from cytotoxic effects [6]. Therefore, our finding indicated wild mungbean 61 exhibited a higher resistance to drought-stress compared with cultivar 70. The distinct phenotypic and physiological responses indicated wild mungbean 61 and cultivar 70 are two contrasting genotypes for drought tolerance. As reported in soybean, the wild germplasm possessed valuable candidate genes which made the plants more drought-tolerant [38]. Thus, we further compared the gene expression changes and DNA methylation patterns alterations from the genome scale.

From the transcriptomic data, when wild mungbean 61 and cultivar 70 were compared, 2859 DEGs were detected. The number increased to 3121 in the comparison of D70 vs D61. However, it was obvious in D61 vs C61 and D70 vs C70, the DEGs were 1117 and 185 respectively. The data indicated the inherent genetic variations existed between these two contrasting genotypes. Interestingly, after drought stress there were more DEGs in D61 vs C61, while less DEGs in D70 vs C70. Study in onion also found that in drought-tolerant genotype more DEGs detected than in drought-sensitive genotype [41]. Among the DEGs, after drought stress, similar level of up-regulated and down-regulated DEGs were found in drought-tolerant onion, and more down-regulated DEGs were found in drought-sensitive genotype [41]. Our study found more down-regulated DEGs than up-regulated in D61 vs C61, whereas opposite response was observed in D70 vs C70. Corresponding to this, after drought stress, more hypermethylated DMRs in wild mungbean 61 were detected and more hypomethylated DMRs in cultivar mungbean 70 were found. Our findings were consistent with the commonly accepted regulative relationship that DNA methylation is negatively associated with gene expression [42, 43]. The pathway enrichment of DEGs suggested that most of them were enriched in carbohydrate metabolism and signal transduction pathways. Carbohydrate metabolism were reported plays important roles in response to drought stress, the starch and sucrose metabolism is correlated with turgor pressure maintenance [44, 45]. In addition, the signal transduction pathway was also found participated in the drought stress response [44, 46].

Our study found that in C61 and C70, the proportion of methylated CG, CHG and CHH was around 24, 28, and 48% respectively, which was similar to the results of previous study using the same mungbean material as cultivar 70 [47]. After drought treatment, genome-wide changes of CHH methylation were relatively bigger than CG and CHG, with increased CHH (from 48.1 to 48.9%) observed in D61 vs C61, but decreased CHH (from 47.9 to 46.9%) was found in D70 vs C70. Similar to our finding in D61 vs C61, in cotton, more significant changes of CHH rather than CG and CHG were found after drought stress, and CHH tended to be hypermethylated [48]. In apple, a slight increased CG and CHG methylation proportions as well as a decreased CHH proportions were revealed after water deficit [29], which was consistent to our report in D70 vs C70. Further investigation confirmed the increase of CHH in D61 vs C61 was mainly contributed by 5UTR, exon, 3UTR, and repeat regions, while the decrease of CHH in D70 vs C70 was mainly contributed by promoter, intron, and repeat regions. The preference of methylation status alterations in CHH suggested asymmetric CHH changes were dynamic and probably associated with external environments [48, 49].

We further compared the epigenetic changes from genome-scale and analyzed the interactions between DNA methylation and gene expression. Our data showed high expression in gene body tended to have lower-level methylation, and non-expressed genes had higher level methylation. Vice versa, the highest methylation levels in gene body showed the lowest expression levels. Earlier report in *Arabidopsis thaliana* indicated loss of methylation in gene body promoted transcription of genes [50]. However, studies in rice and apple revealed that gene body methylation was commonly positively associated with gene expression [29, 51]. Previous study reported that different DNA sequence context and different genomic regions showing varied effect on gene expression [52]. In our study, in gene body the moderately CG methylated genes showed the highest level of expression, which is consistent with the reports in poplar [53, 54]. As is known, DNA methylation in promoters is likely to impede transcription [50]. In our study, in promoter, the highest methylation levels also showed the lowest expression levels in all three DNA sequence contexts in the wild mungbean 61, however, only in CG and CHG for cultivar mungbean 70. Similarly, CHH methylation levels in apple was found positively assiciated with gene expression, which was different from CG and CHG [29]. In addition, for CHH methylation, the unexpressed genes showing the highest methylation level was observed in promoter in the wild mungbean 61, but not in promoter in cultivar mungbean 70. Altogether, based on the facts that CHH mehylation and gene expression in promoter in mungbean 70 was significantly different from others, it was obvious that main variations between wild mungbean 61 and cultivar mungbean 70 existed in CHH methylation in promoter. Previously, we also found the preference of methylation status alterations in CHH in both D61 vs C61 and D70 vs C70. Taken together, our finding suggests asymmetric CHH contexts were more dynamic and prone to be altered by environmental factor changes and genotypic variations. CHH methylation, which is maintained by CMT2 through RdDM, has been proven to be dynamic and play important roles in regulating gene expression during seed development, germination, and early plant life [55–57]. In rice, in response to desiccation and salinity stresses, methylation levels of CHH showed the most variation between different genotypes, suggesting the important role of CHH in abiotic stress response [58]. Further analysis of the correlation between DMRs and DEGs indicated in both D61 vs C61 and D70 vs C70, the DMRs in gene body was significantly negatively correlated with DEGs. However, no significant difference was detected between DMRs and DEGs in promoter. Our results indicated DNA methylation partially contributed to gene expression regulation. In addition, in the poplar salt stress study, few DEGs were identified as different methylation genes, which suggests that the impact of DNA methylation on gene expression is limited [53]..

## Conclusions

Compared with cultivar 70, wild mungbean 61 exhibited a higher resistance to drought-stress, reflecting in lower level of MDA and higher levels of SOD, POD, and CAT. Transcriptomic analysis indicated when drought-treated 61 and 70 compared with their controls, more down-regulated DEGs than up-regulated was found, which was opposite in D70 vs C70. Corresponding to this, after drought stress, more hypermethylated DMRs in 61 were detected, with more hypomethylated DMRs in 70. In addition, we found the main variations between the two contrasting genotypes existed in CHH methylation in promoter. Coincidently, the methylation status alterations in D61 vs C61 and D70 vs C70 also fell in CHH sequence context. Further analysis of the correlation between DMRs and DEGs indicated in both D61 vs C61 and D70 vs C70, the DMRs in gene body was significantly negatively correlated with DEGs.

## Methods

### Plant material and drought treatment

The mungbean cultivar ‘Zhonglu 1’ (germplasm accession no. VC1973A, named 70 in this study) and wild type (germplasm accession no. JP226873, named 61 in this study) were used in this study. The seeds were kindly provided by Dr. Suk-Ha Lee from Department of Plant Science and Research Institute for Agriculture and Life Sciences, Seoul National University. The germinated seeds were grown in pots in growth chamber of Qingdao Agricultural University (Qingdao, Shandong, China) at 24 ± 2 °C day and 17 ± 2 °C night under the photoperiod of 18/6 h day/night. Plants were divided into four groups: a) well-watered 70 (C70); b) drought-stressed 70 (D70); c) well-watered 61 (C61); d) drought-stressed 61 (D61), with three biological replicates in each group for physiological parameters determination, with two biological replicates in each group for transcriptome and methylome study. The well-watered groups were irrigated normally to maintain water capacity, and drought-stressed groups were withheld water since the time planted. Seedlings with the same growth stage were selected for sampling. Leaf samples were collected near to V1 stage (fully developed trifoliate at the second node) when relative water content of soil reached to 39% in drought-stressed groups, which was 69% in well-watered control. The relative water content was calculated by fresh weight subtracting dry weight, and then divided by turgid weight subtracting dry weight according to previous report [59]. The collected leaf samples were stored at − 80 °C until used for RNA and DNA extraction.

### Physiological parameters determination

The oxidative stress biomarker and antioxidant-related indicators MDA (Solarbio, BC0025), SOD (Solarbio, BC0175), POD (BC0095), and CAT (Solarbio, BC0205) was detected by using assay kits and with a BioTek Cytation 1 cell imaging multimode reader (BioTek, Winooski, VT, USA). Fresh mungbean leaf tissue was collected and the measurement was performed following the manufacturer’s instructions of Beijing Solarbio Science & Technology Co., Ltd. (Beijing, China).

### RNA isolation, RNA-sequencing and data analysis

Total RNA was extracted using RNAprep Pure Plant Kit (DP441, TIANGEN Biotech). The integrity of isolated RNA was assayed through the RNA 6000 Nano labchip on 2100 Agilent Bioanalyzer (Agilent Technologies, Santa Clara, CA) before RNA-Seq libraries preparation. RNA-Seq libraries were constructed using TruSeq Stranded mRNA LTSample Prep Kit (Illumina, San Diego, CA, USA) according to the manufacturer’s instructions. After quality inspection through 2100 Agilent Bioanalyzer, the prepared RNA-Seq libraries were sequenced on Illumina HiSeq X Ten platform by OE biotech Co., Ltd. (Shanghai, China). The raw reads were filtered and trimmed using Trimmomatic v0.32 [60]. The obtained clean reads were aligned to reference genome using HISAT2 [61]. Fragments Per Kilobase of transcript per Million mapped fragments (FPKM) with Cufflinks were used to calculate the expression levels of each gene, and the read count of each gene were generated by HTSeq [62]. DESeq was used to determine DEGs [63], with *p*-value < 0.05 and |log2 Fold change (logFC)| > 1 setting as the cutoff for significantly DEGs. KEGG pathway enrichment analysis was performed to investigate the biological functions of DEGs using R based on the hypergeometric distribution [40].

### DNA extraction, WGBS, and data analysis

Genomic DNA was extracted using modified CTAB method [64]. DNA concentration was quantified using Qubit DNA BR Assay Kits (Invitrogen, Eugene, OR, USA) according to the manufacturer’s instructions. Totally, 100 ng genomic DNA spiked with 9 ng lambda DNA were sonicated into 200–300 bp fragments with Covaris S220, and then treated with sodium bisulfite using the EZ DNA Methylation-Gold Kit (Zymo Research). The spiked lambda DNA was used as an unmethylated reference for conversion efficiency calculation. The prepared libraries were sequenced on Illumina Novaseq platform by OE biotech Co., Ltd. (Shanghai, China) after quality assessment on the 2100 Agilent Bioanalyzer. Bismark software (version 0.16.3) was used for alignments of reads to a reference genome [65]. Bioconductor package DSS software was used for identification of DMRs [66]. The genes related to DMRs was defined as the genes with gene body region or promoter region have an overlap with the DMRs. GOseq R package was used for Gene Ontology (GO) enrichment analysis of genes related to DMRs [67], and KOBAS software was used to determine the statistical enrichment of DMR-related genes in KEGG pathways [68].

### Quantitative real-time PCR analysis

A total of 1.5 μg RNA was used for reverse transcription to obtain complementary DNA (cDNA) using 5X All-In-One RT MasterMix (abm, China). The primers used for qRT-PCR were list in Additional file 6: Table S1. The reactions for qRT-PCR were performed based on the protocol of ChamQ SYBR Color qPCR Master Mix (Vazyme, Shanghai, China), with two biological and three technical replicates using a CFX96 instrument (Bio-Rad, Hercules, CA, USA). The total amplification volume was 10 μL per reaction, and the conditions for PCR reaction were as follows: 95 °C for 30 s, 35 cycles of 95 °C for 10 s, 53 °C for 30 s, and 72 °C for 30 s, then followed by 65 °C for 5 s and 95 °C for 5 min. The relative gene expression data was calculated using 2^-ΔΔCt^ method.

## Supplementary Information

**Additional file 1: Figure S1.** Heatmap of DEGs in four pairwise comparisons. a C70 vs C61. b D70 vs D61. c D70 vs C70. d D61 vs C61.

**Additional file 2: Figure S2.** Validation of the reliability of RNA-seq data by qRT-PCR. The vertical axis indicates the fold change when drought stressed D61 compared with control C61 (a), and D70 compared with C70 (b); the horizontal axis shows the eight DEGs selected.

**Additional file 3: Figure S3.** Number of differentially methylated regions in D61 vs C61 and D70 vs C70.

**Additional file 4: Figure S4.** DNA methylation levels of DMRs in all CG, CHG, and CHH contexts displayed by violin boxplots in D61 vs C61 (a) and D70 vs C70 (b). Number of DMRs in different regions of the genome in D61 vs C61 (c) and D70 vs C70 (d).

**Additional file 5: Figure S5.** Relationship between DNA methylation and gene expression in C61, D61, C70 and D70 in gene body. Expression profiles of different methylated levels at CG (a), CHG (b) and CHH (c) were investigated. The gene body methylation levels were classified into five groups with group.1st the lowest and group.5th the highest.

**Additional file 6: Table S1.** Primers used for qRT-PCR analysis.

## Data Availability

All related sequencing data is deposited in NCBI Sequence Read Archive (SRA) database with the link of https://www.ncbi.nlm.nih.gov/Traces/study/?acc=PRJNA771920. The bioProject accession is PRJNA771920 and BioSample accessions are from SAMN22349573 to SAMN22349580.

## Abbreviations

DEGs: Differentially expressed genes
DMRs: Differentially methylated regions
KEGG: Kyoto encyclopedia of genes and genomes
SOD: Superoxide dismutase
POD: Peroxidase
CAT: Catalase
MDA: Malondialdehyde
RdDM: RNA-directed DNA methylation
MET1: DNA Methyltransferase 1
CMT3: Chromomethylase 3
DRM1: Domains Rearranged Methyltransferases 1
DRM2: Domains Rearranged Methyltransferases 2
