# Supplementary figures and images for: Transcriptome and methylome changes in two contrasting mungbean genotypes in response to drought stress
Source: BMC Genomics. 2022 Jan 25;23:80. doi: 10.1186/s12864-022-08315-z (PMC8790888; doi:10.1186/s12864-022-08315-z)

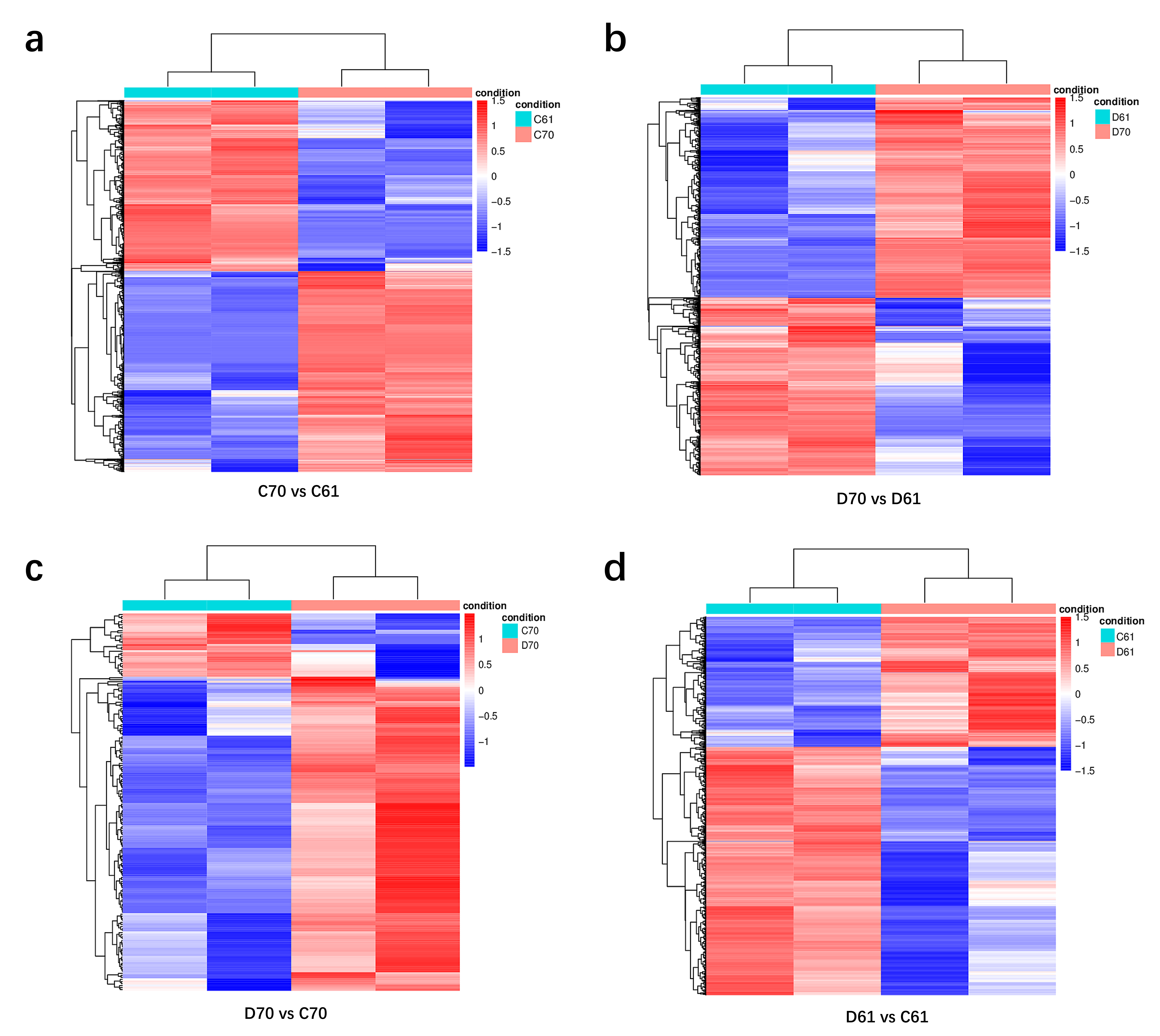

Supplement: Supplementary file 1 — Additional file 1: Figure S1. Heatmap of DEGs in four pairwise comparisons. a C70 vs C61. b D70 vs D61. c D70 vs C70. d D61 vs C61. [file 12864_2022_8315_MOESM1_ESM.png]

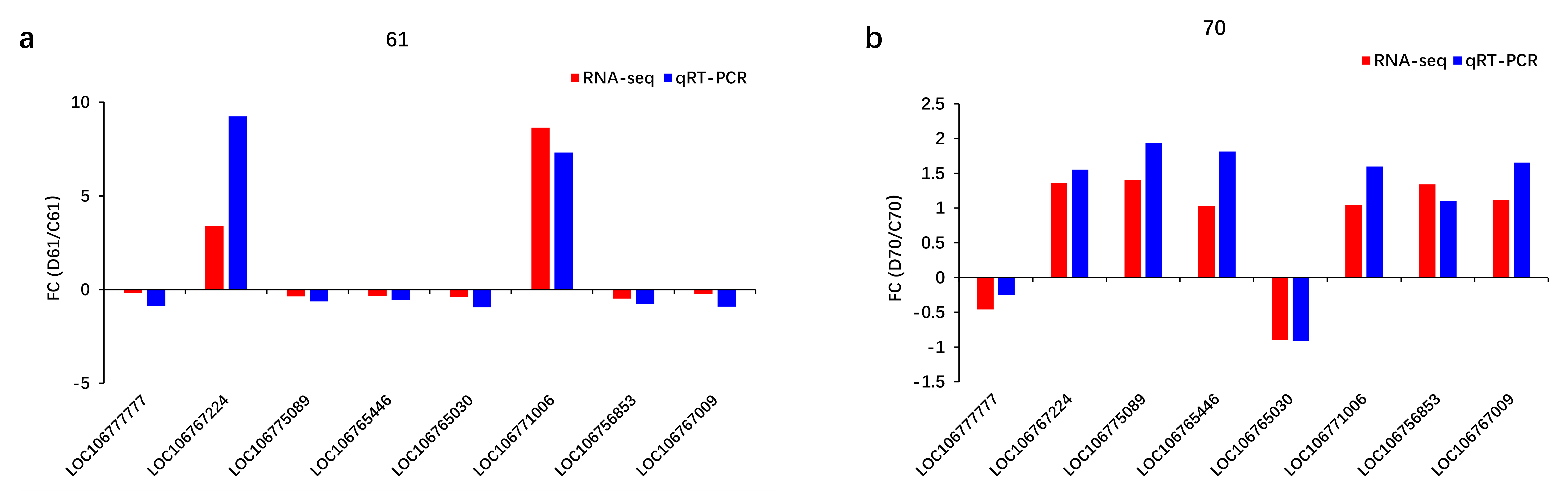

Supplement: Supplementary file 2 — Additional file 2: Figure S2. Validation of the reliability of RNA-seq data by qRT-PCR. The vertical axis indicates the fold change when drought stressed D61 compared with control C61 (a), and D70 compared with C70 (b); the horizontal axis shows the eight DEGs selected. [file 12864_2022_8315_MOESM2_ESM.png]

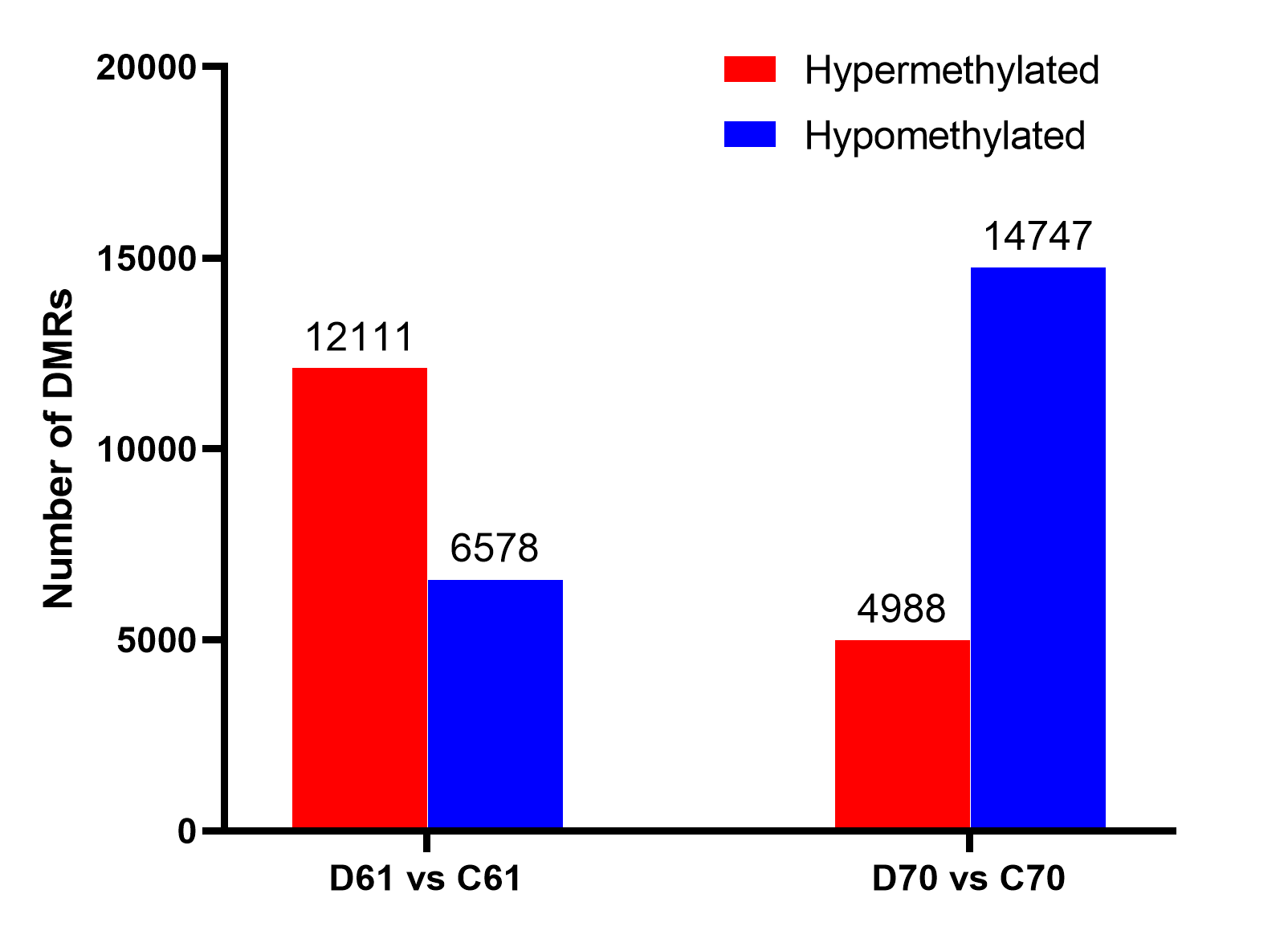

Supplement: Supplementary file 3 — Additional file 3: Figure S3. Number of differentially methylated regions in D61 vs C61 and D70 vs C70. [file 12864_2022_8315_MOESM3_ESM.png]

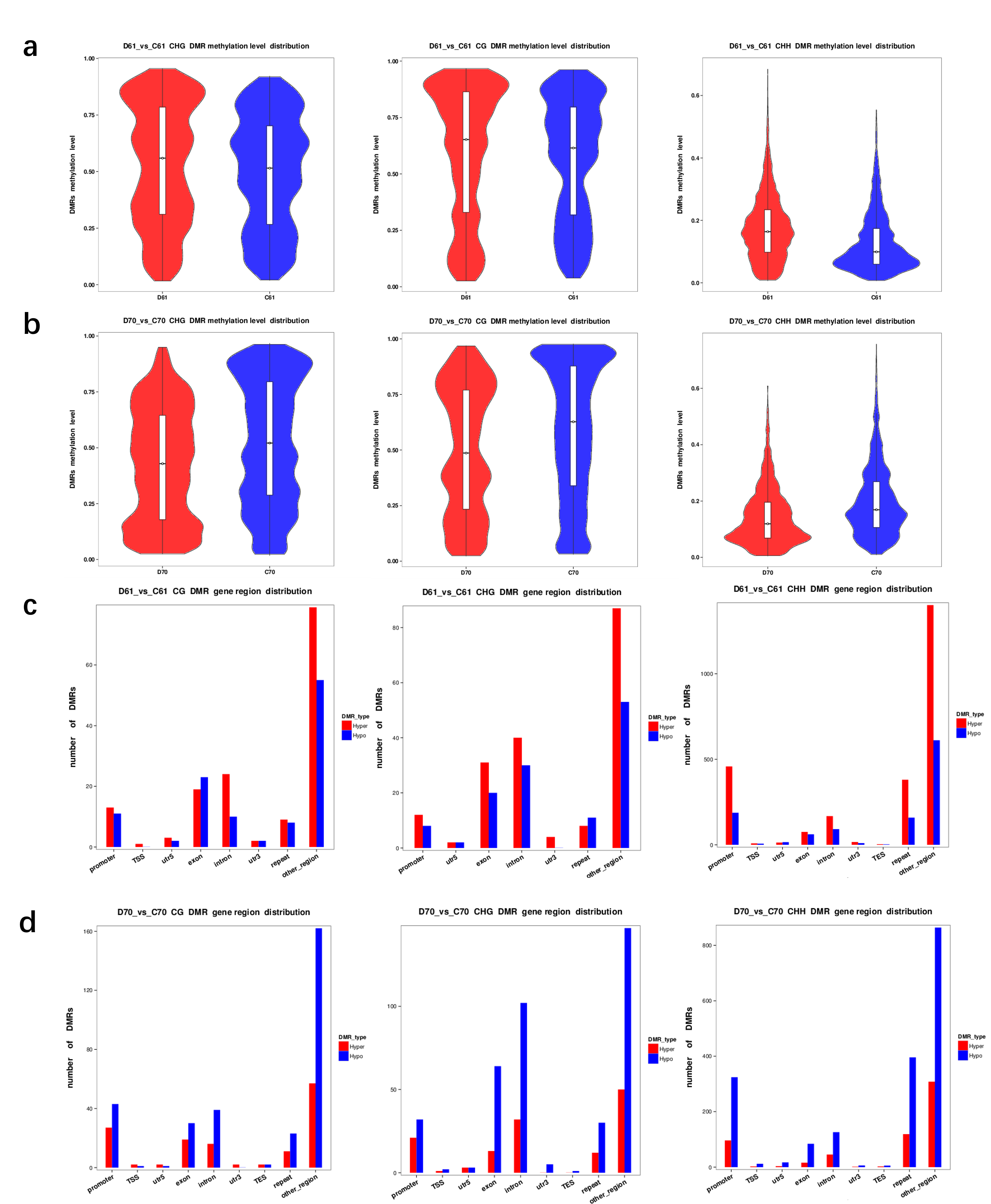

Supplement: Supplementary file 4 — Additional file 4: Figure S4. DNA methylation levels of DMRs in all CG, CHG, and CHH contexts displayed by violin boxplots in D61 vs C61 (a) and D70 vs C70 (b). Number of DMRs in different regions of the genome in D61 vs C61 (c) and D70 vs C70 (d). [file 12864_2022_8315_MOESM4_ESM.png]
