# Supplementary material for: Transcriptome and methylome changes in two contrasting mungbean genotypes in response to drought stress
Source: BMC Genomics. 2022 Jan 25;23:80. doi: 10.1186/s12864-022-08315-z (PMC8790888; doi:10.1186/s12864-022-08315-z)
Supplement: Supplementary file 6 — Additional file 6: Table S1. Primers used for qRT-PCR analysis. [file 12864_2022_8315_MOESM6_ESM.docx]

**Additional file 1: Figure S1.** Heatmap of DEGs in four pairwise comparisons. **a** C70 vs C61. **b** D70 vs D61. **c** D70 vs C70. **d** D61 vs C61.

**Additional file 2: Figure S2.** Validation of the reliability of RNA-seq data by qRT-PCR. The vertical axis indicates the fold change when drought stressed D61 compared with control C61 (**a**), and D70 compared with C70 (**b**); the horizontal axis shows the eight DEGs selected.

**Additional file 3: Figure S3.** Number of differentially methylated regions in D61 vs C61 and D70 vs C70.

**Additional file 4: Figure S4.** DNA methylation levels of DMRs in all CG, CHG, and CHH contexts displayed by violin boxplots in D61 vs C61 (**a**) and D70 vs C70 (**b**). Number of DMRs in different regions of the genome in D61 vs C61 (**c**) and D70 vs C70 (**d**).

**Additional file 5: Figure S5.** Relationship between DNA methylation and gene expression in C61, D61, C70 and D70 in gene body. Expression profiles of different methylated levels at CG (**a**), CHG (**b**) and CHH (**c**) were investigated. The gene body methylation levels were classified into five groups with group.1st the lowest and group.5th the highest.

**Additional file 6: Table S1.** Primers used for qRT-PCR analysis

| **Gene ID** | **Annotation** | **Forward primer (5'-3')** | **Reverse primer (5'-3')** |
| --- | --- | --- | --- |
| VrActin |  | CAGTGTCTGGATTGGAGGCT | GTCCTCGACCACTTGATG |
| LOC106777777 | CYP707A2 | CAAGGACTTGTTGGGTTCGT | GACACTGGGGTTTTCTCCAA |
| LOC106767224 | MYB102 | GGCTATTGCTTCTCGTTTGC | TGTCTGTGTGCCAAGGAGTC |
| LOC106775089 | PCNA | CACCAAGGAGGGTGTGAAGT | TCAACAACAACTGGCAGCTC |
| LOC106765446 | MCM5 | GGTCCGGTTAAGAGGTGACA | TCACGCGTACTGCTATCCTG |
| LOC106765030 | BHLH74 | GCCGTAATGCTGGATGAGAT | ACTGGGGAATGTGTGAGAGG |
| LOC106771006 | NAC031 | CCCAGTGATGAGGAGTTGGT | TTGCATATTTTCGGTCACGA |
| LOC106756853 | GIS | TCATCAGCTTCAACCCATCA | CATCTTCGCCCTCATCATTT |
| LOC106767009 | HMGB6 | CCTCCATCATCGTTCATCCT | AGACGCTTGGCTATTCCAGA |
